# Supplementary material for: Disease Resistant Bouquet Vine Varieties: Assessment of the Phenolic, Aromatic, and Sensory Potential of Their Wines
Source: Biomolecules. 2019 Nov 27;9(12):793. doi: 10.3390/biom9120793 (PMC6995519; doi:10.3390/biom9120793)
Supplement: Supplementary file 1 [file biomolecules-09-00793-s001.pdf]

Supplementary material

Figure S1. Sensory evaluation of monovarietal red wines from the new hybrid grape (HG) varieties. * indicates significance at $p < 0.05$.

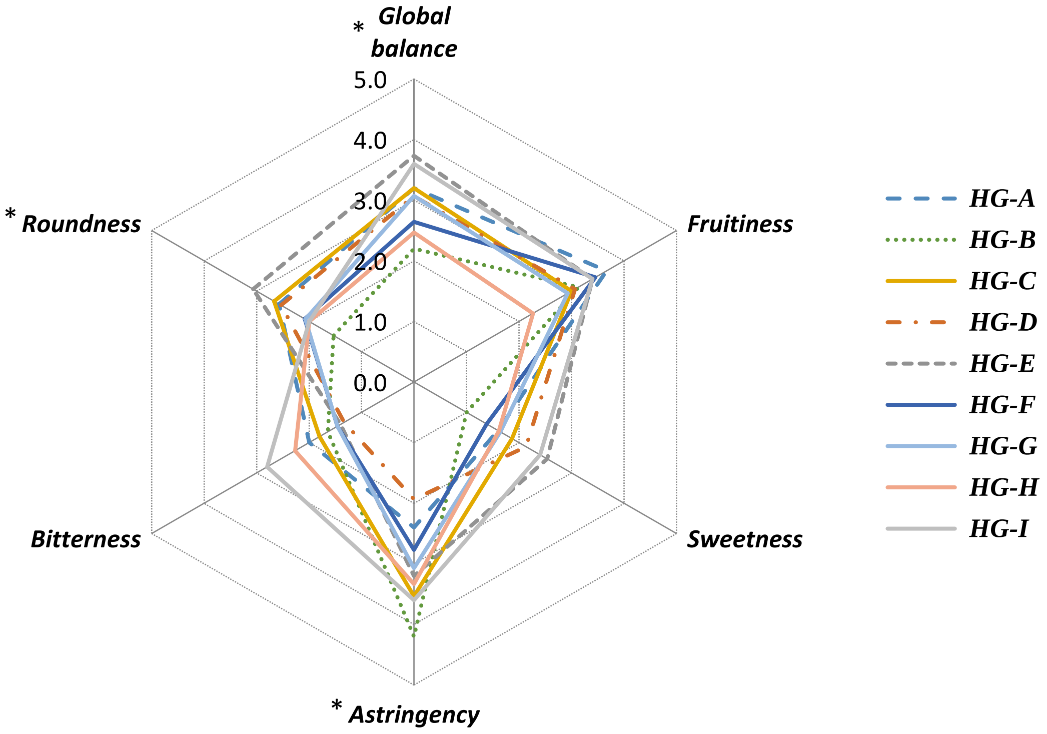

Table S1. Oenological and chromatic parameters of monovarietal red wines from the new hybrid grape (HG) varieties.

	HG-A	HG-B	HG-C	HG-D	HG-E	HG-F	HG-G	HG-H	HG-I
Oenological parameters									
Alcoholic degree (%)	11,6 ± 0,0	12,5 ± 0,0	14,3 ± 0,0	12,9 ± 0,0	16,6 ± 0,0	11,9 ± 0,1	12,8 ± 0,0	11,6 ± 0,0	12,3 ± 0,0
pH	4,0 ± 0,0	3,7 ± 0,0	4,0 ± 0,0	3,6 ± 0,0	4,3 ± 0,0	3,5 ± 0,0	4,0 ± 0,0	3,9 ± 0,0	3,9 ± 0,0
TA (g H_2SO_4/L)	2,7 ± 0,0	3,1 ± 0,0	2,8 ± 0,0	2,8 ± 0,0	3,2 ± 0,0	3,3 ± 0,0	2,5 ± 0,0	3,0 ± 0,0	3,4 ± 0,0
VA (g H_2SO_4/L)	0,4 ± 0,0	0,4 ± 0,0	0,5 ± 0,0	0,3 ± 0,0	0,7 ± 0,0	0,4 ± 0,0	0,4 ± 0,0	0,3 ± 0,0	0,7 ± 0,0
Density (g/mL)	1,0 ± 0,0	1,0 ± 0,0	1,0 ± 0,0	1,0 ± 0,0	1,0 ± 0,0	1,0 ± 0,0	1,0 ± 0,0	1,0 ± 0,0	1,0 ± 0,0
Gluc/Fruc	1,9 ± 0,1	2,6 ± 0,0	3,8 ± 0,0	3,1 ± 0,0	4,7 ± 0,1	2,3 ± 0,1	3,2 ± 0,1	3,2 ± 0,1	1,4 ± 0,1
Malic acid (g/L)	0,2 ± 0,0	0,0 ± 0,0	0,0 ± 0,0	0,0 ± 0,0	0,0 ± 0,0	0,2 ± 0,0	0,0 ± 0,0	0,1 ± 0,0	0,0 ± 0,0
Lactic acid (g/L)	2,0 ± 0,0	1,6 ± 0,0	0,9 ± 0,0	0,7 ± 0,0	1,5 ± 0,0	1,0 ± 0,0	1,0 ± 0,0	1,6 ± 0,0	1,0 ± 0,0
Tartaric acid (g/L)	2,0 ± 0,0	3,0 ± 0,0	1,5 ± 0,0	2,3 ± 0,0	2,2 ± 0,0	2,3 ± 0,0	1,3 ± 0,0	2,0 ± 0,0	1,1 ± 0,0
Chromatic parameters									
CI (AU)	1,1 ± 0,0	1,1 ± 0,0	1,2 ± 0,0	0,5 ± 0,0	1,7 ± 0,0	0,9 ± 0,0	0,6 ± 0,0	1,3 ± 0,0	1,7 ± 0,0
Hue (AU)	0,7 ± 0,0	0,6 ± 0,0	0,6 ± 0,0	0,7 ± 0,0	0,6 ± 0,0	0,6 ± 0,0	0,8 ± 0,0	0,6 ± 0,0	0,8 ± 0,0
% yellow	37,5 ± 0,3	33,7 ± 0,5	34,0 ± 0,4	37,5 ± 0,4	33,8 ± 0,3	35,3 ± 0,9	39,5 ± 0,1	32,0 ± 0,3	36,6 ± 0,1
% red	51,1 ± 0,9	55,1 ± 0,8	54,4 ± 0,3	56,4 ± 0,6	52,4 ± 0,3	55,5 ± 1,4	51,3 ± 0,2	56,8 ± 0,4	48,7 ± 0,2
% blue	11,4 ± 0,1	11,2 ± 0,3	11,6 ± 0,2	6,1 ± 0,1	13,8 ± 0,2	9,2 ± 0,3	9,2 ± 0,1	11,2 ± 0,1	14,7 ± 0,1

Table S2. Bibliographic data about fruity aroma profile by ester families for monovarietal wines made from international red grape varieties (Cabernet Sauvignon, Merlot, Syrah).

Bibliographic reference	Wine characteristics			Ethyl esters of straight-chain fatty acids[a]	Higher alcohol acetates[a]	Ethyl branched acid esters[a]
	Geographical origin	Vintage	Grape variety			
Antalick et al., 2012	France, Switzerland	2007 - 2009	CS, Merlot[b]	1150	578	65
Antalick et al., 2015	Australia	no specified	CS	1258 - 1434	528 - 654	52 - 74
			Syrah	1470 - 1553	1403 - 1501	52 - 80
Arcari et al., 2017	Brazil	2008 - 2013	Merlot	370 - 3814	39 - 116	41 - 147
Gammacurta et al., 2014	Bordeaux (France)	2011 - 2012	CS	546 - 4204	710 - 7569	25 - 105
			Merlot	672 - 1836	298 - 878	49 - 81
González-Centeno et al., 2016	France, Italy, United States	2013	CS	562 - 1531	310 - 718	80 - 174

CS, Cabernet Sauvignon. [a] Results expressed in µg/L wine. [b] Results are a mean of ester content of both grape varieties

Table S3. Fruity volatile profile of monovarietal red wines from the new hybrid grape (HG) varieties. For each individual ester, lower case letters *a−i* show significant differences among hybrid grape varieties ($p < 0.05$).

	HG-A	HG-B	HG-C	HG-D	HG-E	HG-F	HG-G	HG-H	HG-I
Ethyl esters of straight-chain fatty acids									
Ethyl propanoate	247,6 ± 3,5 *f*	332,0 ± 2,1 *c*	318,3 ± 3,6 *d*	221,2 ± 7,6 *g*	346,0 ± 5,2 *b*	276,6 ± 3,6 *e*	212,2 ± 1,3 *g*	184,2 ± 2,1 *h*	436,0 ± 7,9 *a*
Ethyl butyrate	101,4 ± 0,2 *b*	138,3 ± 1,2 *a*	84,8 ± 0,0 *d*	86,1 ± 1,6 *d*	135,6 ± 1,3 *a*	78,6 ± 1,5 *e*	57,6 ± 0,2 *g*	59,9 ± 0,5 *f*	98,1 ± 1,2 *c*
Ethyl hexanoate	130,5 ± 0,3 *d*	208,3 ± 1,6 *a*	127,2 ± 0,1 *d*	129,8 ± 2,8 *d*	110,5 ± 0,5 *e*	149,7 ± 2,5 *b*	148,6 ± 0,2 *b*	128,0 ± 0,7 *d*	136,2 ± 2,8 *c*
Ethyl octanoate	196,5 ± 1,3 *a*	199,5 ± 1,0 *a*	105,2 ± 0,7 *e*	130,2 ± 3,4 *b*	107,3 ± 0,6 *e*	113,6 ± 0,7 *d*	86,5 ± 0,6 *f*	122,9 ± 0,1 *c*	135,3 ± 4,1 *b*
Ethyl decanoate	65,9 ± 1,0 *a*	49,5 ± 1,1 *b*	45,6 ± 0,5 *c*	40,5 ± 0,8 *d*	38,4 ± 0,3 *d*	35,1 ± 0,4 *e*	24,2 ± 1,1 *f*	41,1 ± 1,4 *d*	40,8 ± 1,7 *d*
Dodecanoate d'éthyle	3,2 ± 0,2 *b*	1,7 ± 0,1 *e*	2,0 ± 0,1 *d*	5,4 ± 0,1 *a*	1,6 ± 0,0 *e*	2,7 ± 0,1 *c*	0,6 ± 0,1 *g*	1,2 ± 0,1 *f*	4,7 ± 0,4 *a*
Higher alcohol acetates									
Propyl acetate	30,9 ± 0,7 *e*	54,2 ± 0,3 *b*	35,9 ± 0,2 *d*	15,6 ± 0,7 *g*	72,9 ± 0,5 *a*	42,6 ± 1,1 *c*	18,0 ± 0,3 *f*	11,0 ± 0,1 *h*	42,3 ± 1,8 *c*
Isobutyl acetate	27,6 ± 0,2 *d*	41,3 ± 0,0 *c*	24,9 ± 0,1 *e*	22,0 ± 0,7 *g*	48,3 ± 0,2 *b*	23,2 ± 0,3 *f*	28,1 ± 0,2 *d*	24,2 ± 0,0 *ef*	51,1 ± 0,8 *a*
Butyl acetate	1,1 ± 0,0 *e*	0,9 ± 0,1 *f*	2,3 ± 0,1 *b*	1,1 ± 0,1 *e*	3,1 ± 0,2 *a*	1,1 ± 0,1 *e*	1,3 ± 0,1 *d*	1,0 ± 0,0 *e*	1,6 ± 0,0 *c*
Isoamyl acetate	199,0 ± 1,8 *d*	175,9 ± 1,7 *e*	316,9 ± 3,2 *a*	186,2 ± 2,9 *de*	265,6 ± 3,6 *b*	173,2 ± 16,1 *e*	306,3 ± 0,4 *a*	267,5 ± 0,3 *b*	233,7 ± 1,3 *c*
Ethyl esters branched acids									
Ethyl isobutyrate	78,8 ± 0,1 *e*	181,5 ± 0,1 *b*	73,3 ± 0,7 *f*	74,0 ± 0,1 *f*	86,4 ± 0,9 *d*	165,5 ± 3,2 *c*	64,7 ± 0,3 *g*	79,4 ± 0,2 *e*	402,4 ± 3,0 *a*
Ethyl 2-methylbutanoate	8,7 ± 0,1 *h*	16,7 ± 0,0 *c*	11,8 ± 0,1 *f*	12,4 ± 0,1 *e*	10,2 ± 0,1 *g*	19,7 ± 0,0 *b*	8,5 ± 0,0 *i*	16,0 ± 0,0 *d*	47,6 ± 0,4 *a*
Ethyl 3-methylbutanoate	8,8 ± 0,1 *f*	30,7 ± 0,2 *b*	13,3 ± 0,0 *d*	12,9 ± 0,2 *d*	11,0 ± 0,5 *e*	21,8 ± 0,9 *c*	8,5 ± 0,0 *f*	11,2 ± 0,1 *e*	50,1 ± 0,5 *a*

All results are expressed in µg/L wine. HG, hybrid grape. Letters a-i following the values in each row show the significant differences among hybrid grape varieties (p < 0.05).
